# Jew and Gentile

**Published:** 1879-09

**Authors:** 


### JEW AND GENTILE.

Israelites live and flourish in all countries and in all climes, beyond any other race and nation; the reason is, the God of their fathers promised it, even before they became a nation. At the same time the Ruler of the world uses instrumentalities, and accomplishes his designs by the operation of natural laws. A very large number of the observances imposed through Moses and Aaron had two main ends in view—to prove their obedience and to preserve their lives—to enable them to live healthily, happily and long. The result is, that of all the peoples who have ever lived the Jew only retains his name, and nation, and lineage. Hence their existence to-day is the greatest miracle connected with the Christian religion, for it is founded on the Bible, and the simple existence of the Jews as a people demonstrates the fact that the Bible is the Word of God.

Dr. Nenfville, of Frankfort — the birth-place of the Rothschilds—states that, in round numbers, the average duration of the life of the Israelites in that city is forty-nine years; that of the rest of the population is thirty-seven years.

During the first five years of life Jewish deaths are but little over one half as many as Christian.

One fourth of Christian children die before they are seven years old, while three fourths of the Jews live to the age of twenty-eight years.

